# The Auxiliary Role of Cardiac Magnetic Resonance Feature-Tracking Parameters in the Differentiation between Cardiac Amyloidosis and Constrictive Pericarditis

**DOI:** 10.1155/2021/2045493

**Published:** 2021-10-23

**Authors:** Sanaz Asadian, Mahta Farzin, Faezeh Tabesh, Nahid Rezaeian, Hooman Bakhshandeh, Leila Hosseini, Yaser Toloueitabar, Mohammad Mehdi Hemmati Komasi

**Affiliations:** ^1^Rajaie Cardiovascular Medical and Research Center, Iran University of Medical Sciences, Tehran, Iran; ^2^Iran University of Medical Sciences, Tehran, Iran; ^3^North Khorasan University of Medical Sciences, Bojnurd, Iran

## Abstract

**Objectives:**

Cardiac amyloidosis (CA) and constrictive pericarditis (CP) are described as the differential diagnoses of restrictive hemodynamic alterations of the heart. We aimed to explain cardiac magnetic resonance (CMR) imaging findings (especially feature tracking (FT)) of CA and CP cases and compare them with healthy controls. Moreover, we evaluated the role of biventricular FT parameters in differentiating CA from CP.

**Methods:**

Thirty-eight patients who underwent CMR between February 2016 and January 2018 with the ultimate diagnosis of CA (19 patients) or CP (19 patients) were enrolled. We included biopsy-proven light-chain amyloidosis patients. The data of 28 healthy controls were utilized for comparison. The patients were followed up for 8–23 months to register mortality and their surveillance. All CMR morphological and functional data, including FT parameters, were recorded and analyzed.

**Results:**

Of only 13/19 (68.4%) CA patients who had the follow-up data, 11/13 (84.6%) died. One of The CP patients (5.3%) expired during the follow-up. Significant between-group differences were noted concerning the biventricular ejection fraction as well as global longitudinal, circumferential, and radial strain values (*Ps* < 0.001). The left ventricular (LV) global longitudinal strain (GLS) ≤10% was detected in 13/19 (68.4%) of the CA and 1/19 (5.3%) of CP cases (*P* < 0.001). A significant difference between the mean value of the LVGLS and LV global circumferential strain (GCS) of the basal LV level compared to the mid and apical levels was observed (*Ps* < 0.001) in the CA patients. The differences between the mean LVGLS and the GCS measures of the mid and apical LV levels were not significant (*P*=1 and *P*=0.06, respectively).

**Conclusions:**

In our study, CA and CP severely disrupted ventricular strains. Biventricular GLS was meaningfully lower in the CA subjects. Therefore, strain analysis, especially in the longitudinal direction, could be helpful to differentiate CA from CP.

## 1. Introduction

Cardiomyopathies, which are commonly classified based on structural and hemodynamic criteria, are subdivided into dilated, hypertrophic, restrictive (RCM), arrhythmogenic, infiltrative, and ischemic types [1]. RCM is an uncommon type of cardiomyopathy resulting in myocardial stiffness and impaired ventricular filling [2]. Its pathophysiologic basis may be hereditary, acquired, or a combination of both.

One of the chief causes of RCM is cardiac amyloidosis (CA). Amyloidosis is a multisystem disorder in which an unstable and misfolded protein (amyloid) aggregates in different organs [1]. The 2 main types of amyloidosis that affect the heart are light-chain and transthyretin amyloidosis. Amyloid infiltration of the heart results in the thickening of the myocardium and diastolic dysfunction, which ultimately leads to heart failure. The prognosis is largely determined by the occurrence and extent of myocardial involvement [3–6].

A traditional well-known differential diagnosis for CA is constrictive pericarditis (CP). Cardiac surgeries and inflammatory/infectious processes are among the most common causes of CP. Both CA and CP present insidiously and have many imaging features in common [7]. The distinction between these 2 conditions is critical because CP is a curable disease with cardiac surgery, whereas the therapeutic options for CA are more challenging [8].

Cardiac magnetic resonance (CMR) imaging can assist in the differentiation of CA from CP [2, 8]. Utilizing its tissue-characterization capability consisting of late gadolinium enhancement (LGE) study, CMR may play a fundamental role in the diagnosis and treatment guidance in CA. Moreover, it can limit the use of endomyocardial biopsy [9]. The tissue-characterization property of the CMR is helpful in the diagnosis of CP by demonstrating the thickening, edema, and enhancement of the pericardium. The conflict occurs in CP cases without the typical morphological imaging findings which hemodynamically mimic CA [10–12].

Recently developed CMR methods, consisting of mapping techniques and feature tracking (FT), contribute to the diagnosis of variable cardiomyopathies. Noncontrast T1 mapping is a highly accurate tool for the detection of the CA (especially AL subtype) with even more sensitivity comparing with the LGE images. Increased myocardial T1 value may be an indicator of amyloid deposition [13].

FT-CMR method has diagnostic potential in many cardiac disorders such as the different types of cardiomyopathies. This technique is contrast-free and is valuable in patients with the limitation in the administration of the gadolinium-based agents [14–18].

Speckle-tracking echocardiographic studies have demonstrated that strain values help differentiate between CA and CP. Patients with CP have markedly abnormal circumferential deformation with relative sparing of longitudinal strains, whereas CA is associated with abnormal longitudinal mechanics [8]. FT-CMR is a postprocessing technique that is widely used to assess global and regional myocardial function; however, there are scarce data on the role of FT-CMR in differentiating CP from CA [19].

In this study, we investigated CMR characteristics in 2 groups of CA and CP patients. We also compared the 2 groups with healthy controls. Ultimately, we determined FT-CMR capability in differentiating these 2 disorders.

## 2. Methods

### 2.1. Study Population

This retrospective study enrolled all patients who were referred to the imaging department of Rajaie Cardiovascular Medical and Research Center for CMR between February 2016 and January 2018 who were ultimately diagnosed with CA or CP. Nineteen patients with CA and 19 cases of CP were included. Moreover, the CMR data of 28 healthy controls with no signs or symptoms of a cardiac disease without any cardiovascular risk factors were utilized for comparison. Written informed consent was obtained from all the participants and in the case of death from their families. The study was approved by the Ethics Committee of Iran University of Medical Sciences.

All the CA patients had highly suspicious clinical and echocardiographic findings in addition to histologically proven (positive Congo red staining of endomyocardial, abdominal fat, renal, rectal, or bone marrow biopsies) amyloid light-chain amyloidosis. Moreover, CMR findings were characteristics of CA [20]. All our CP patients had typical imaging findings of the disease and underwent pericardiectomy during the follow-up, which confirmed the diagnosis.

All patients suffering from significant arrhythmia, undesirable CMR image quality, or inappropriate cine images for FT analysis were excluded. The follow-up continued for 8–23 months.

### 2.2. Cardiac Magnetic Resonance Imaging Protocol

**Figure 1:**
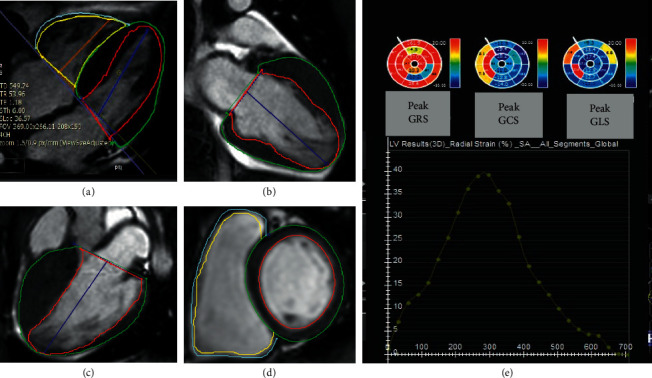
TheCMR feature tracking method for the analysis of myocardial strain. (a–d) Four-, two-, and three-chamber as well as short-axis cine functional images with defined endocardial and epicardial borders for strain analysis by feature-tracking cardiac magnetic resonance method. (e) Bull's eye plot depicts peak segmental longitudinal, circumferential, and radial strain values.

A 1.5 T MRI machine (MAGNETOM Avanto, Siemens Healthcare, Erlangen, Germany) utilizing a vendor-supplied body surface coil was used to perform CMR studies. After axial, coronal, and sagittal localizer images were obtained, 2-, 3-, and 4-chamber, as well as short-axis cine steady-state free precession (SSFP) images, were acquired (slice thickness = 8 mm, field of view = 300 mm, no interslice gap; repetition time/echo time = 3−4/1.2 ms, imaging matrix = 156 × 192, voxel size = 1.9 × 1.6 × 7 mm, and “reported” repetition time (TR)≈31.5 ms) (Figure 1). Axial, coronal, and sagittal T1-weighted images were taken for all the CP patients to assess the pericardial thickness. The LGE sequence was obtained 10–15 minutes after the injection of 0.15 mmol/kg of Dotarem (gadoterate meglumine, Guerbet, Roissy CdG, France) in the same views as the functional images. LGE images were acquired using 2D phase-sensitive inversion recovery (PSIR) Turbo FLASH sequences (slice thickness = 8 mm, TR = 700 ms, time to echo (TE) = 5.4 ms, and flip angle = 25°).

The TI scout sequence was taken at the mid-ventricular level in the short-axis plane (slice thickness = 8 mm, TR = 20 ms, TE = 1.2 ms, flip angle = 50°, and produced with 20 ms increments from 85 to 805 ms). The inversion time applied for the LGE image was the time to the complete nulling of the myocardial signal at the TI scout sequence. LGE sequences were performed 10 minutes after TI scout image.

### 2.3. Image Interpretation

#### 2.3.1. Blood Pool/Myocardial Nulling

On the TI scout sequence, the normal order of nulling is as follows: first, the contrast containing blood pool nulls, followed by the myocardium and the spleen. The nulling pattern was considered abnormal whenever the mentioned order was deranged [21].

#### 2.3.2. LGE Assessment

**Figure 2:**
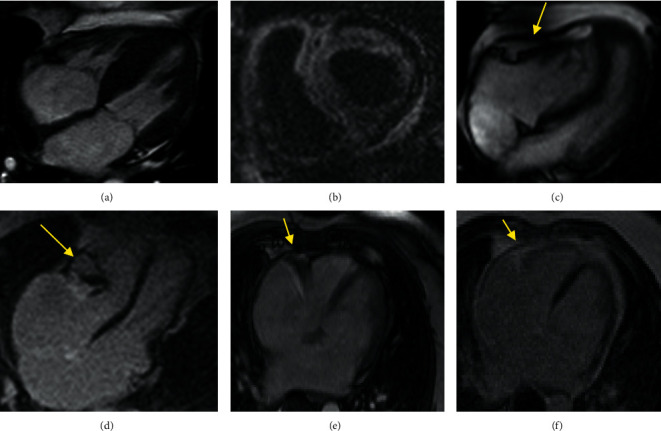
CMR findings in a patient with CA and CP. (a) Four-chamber cine function shows LV hypertrophy and thickened IAS in a CA patient. (b) Short-axis LGE depicts transmural LV and RV GD enhancement in CA. (c) Four-chamber cine function shows pericardial thickening (yellow arrow) in a CP patient. (d) LGE image in four-chamber view shows significant localized pericardial thickening and calcification (yellow arrow). (e, f) Localizer and LGE images of a 49-year-old woman with a history of shortness of breath and palpitations from three months ago. The patient had restrictive physiology on echocardiography. For further evaluation, CMR was performed, which showed restrictive physiology with normal pericardial thickness (arrow), and notably, a moderate reduction in the strain values (GLS: −14.01%), which was more in favor of CP. Hemodynamic finding in invasive angiography was an indicator of constriction. Finally, CP with normal pericardial thickness was confirmed during surgery.

The LGE pattern was divided into no enhancement, subendocardial, and transmural based on the visually assessed enhancement characteristics of our CA patients (Figure 2).

#### 2.3.3. Myocardial Deformation Analysis

**Figure 3:**
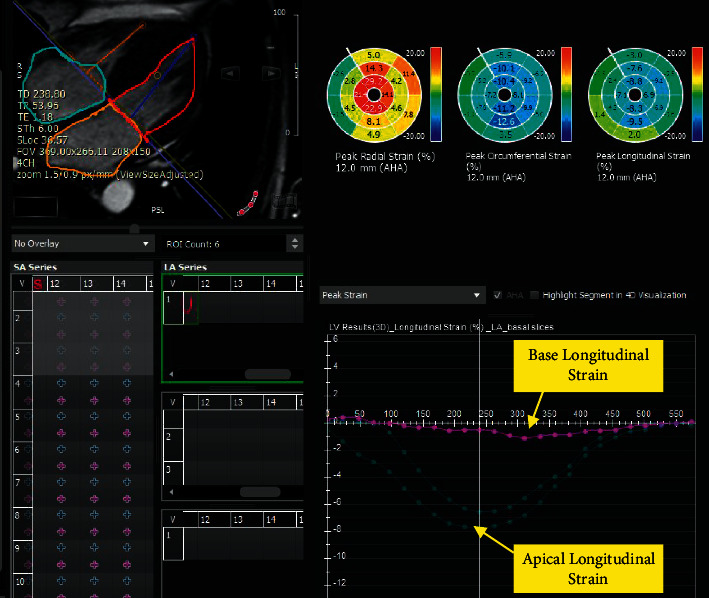
LV longitudinal strain in base, mid, and apical levels in a patient with CA depicts apical sparing with the significant reduction of strain value in the basal level. LV: left ventricle; CA: cardiac amyloidosis.

The strain values were calculated utilizing the cvi42 (Circle Cardiovascular Imaging, Calgary, Alberta, Canada) version 5.6.2 (634). The endocardial and epicardial borders of both ventricles were delineated manually at the end-diastolic frame in 2-, 3-, 4-chamber views and all short-axis stacks for the left ventricle (LV) as well as 4-chamber and short-axis images for the right ventricle (RV). Three-dimensional LV and 2D RV strain values were extracted after the propagation of the contours during the entire cardiac cycle. For both ventricles, the absolute values of the global longitudinal strain (GLS), the global circumferential strain (GCS), and the global radial strain (GRS) were assessed. Furthermore, in CA patients, GLS was evaluated in the LV basal, mid, and apical levels (Figure 3). All the patients were stable hemodynamically with euvolemic status during the CMR examination.

#### 2.3.4. Other Assessments

Left and right atrial (LA and RA) areas, the LV mass index, and the interatrial septal thickness were measured in the 4-chamber view at the end of cardiac systole. The presence of any pericardial or pleural effusion, as well as ascites, was registered. The measurements were done by an expert cardiologist with 5.5 years of experience in the field of cardiac imaging, who was blinded to the study subjects' data.

### 2.4. Follow-Up

All the patients were followed up by medical file reviewing and telephone interviews for 8–23 months to assess the mortality rate. The cardiovascular events were collected by an independent cardiologist blinded to the patient's data and FT values.

### 2.5. Statistical Analysis

The analyses were performed utilizing SPSS software, version 22.00. Normally distributed continuous variables were described as the mean ± the standard deviation (SD), while categorical variables were expressed as frequencies and percentages. The Kolmogorov–Smirnov test was utilized for the evaluation of the normality of the distribution of the numerical variables. The *χ*^2^ test was applied to compare the ratio of the patients with LVGLS ≤10% in the CA and CP groups. For the comparison of the variables between our 3 study groups, the ANOVA test was employed. To modulate the effect of age between nonmatched groups, the analysis of covariance (ANCOVA) test was applied. Then, the post hoc Bonferroni test was utilized to reveal between-group differences. Moreover, a 2-independent sample *t*-test was applied for the intergroup comparisons of quantitative variables. A repeated-measures ANOVA test was performed to evaluate the differences between 3D LV strains at basal, mid, and apical levels in the CA subjects. A cutoff value of 0.05 was considered for the *P* value to mention statistically significant results.

## 3. Results

The study population consisted of 66 subjects: 19 patients with CA (68.4% male, mean age = 57 ± 10 y), 19 cases with CP (73.7% male, mean age = 51 ± 17.5 y), and 28 healthy controls (50% male, mean age = 31 ± 4 y).  Table 1 demonstrates the demographic and CMR values of the study population.

The LV myocardial fibrosis pattern was predominantly transmural in 15/19 (78.9%), predominantly subendocardial in 2/19 (10.5%), and mixed transmural and subendocardial in 2/19 (10.5%) of the CA group.

Only 13/19 (68.4%) CA patients had the follow-up. The mortality rate was 11/13 (84.6%). One of the CP patients (5.3%) died during the follow-up.

Significant differences were found in the mean values of the interatrial septal thickness, the LV mass index, and the biatrial areas between the CA and the healthy controls (all *Ps* < 0.001).

The one-way ANOVA and ANCOVA tests revealed significant differences between our 3 study groups concerning the LV ejection fraction (EF) (*F* [2, 63] = 42.75, *P* < 0.001), EF (*F* [2, 63] = 26.24, *P* < 0.001), LVGLS (*F* [2, 63] = 105.52, *P* < 0.001), LVGCS (*F* [2, 63] = 42.06, *P* < 0.001), LVGRS (*F* [2, 63] = 30.76, *P* < 0.001), RVGLS (*F* [2,63] = 16.49, *P* < 0.001), RVGCS (*F* [2, 63] = 63.96, *P* < 0.001), and RVGRS (*F* [2,63] = 46.99, *P* < 0.001).

The results of the post hoc Bonferroni test to reveal between-group differences are demonstrated in Table 2.

LVGLS ≤10% was detected in 13/19 (68.4%) of the CA group and 1/19 (5.3%) of the CP group. The difference between these groups considering LVGLS ≤10% was significant (*P* < 0.001).

A significant difference in the mean value of the LVGLS of the basal LV level compared with mid and apical levels was detected (mean differences = −7.54 and −7.11; *Ps* < 0.001) in the CA population. The differences between the mean LVGLS measures of the mid and apical LV levels were not significant (mean difference = 0.42; *P*=1.00). Similarly, the LVGCS value at the basal ventricular level was significantly lower than that at the mid and apical levels. The mean differences were −5.56 and −3.98, respectively (*Ps* < 0.001). The difference between the mean GCS value of the mid and apical LV levels was not meaningful (mean difference = 1.57; *P*=0.06). The comparison of the mean GRS value between the 3 LV levels demonstrated a significant difference between the radial strain measures of the apical and basal as well as mid-ventricular levels (mean differences = 18.87 and 17.47; *Ps* < 0.001). The difference in the mean GRS value between the basal and mid-LV levels was not significant (mean difference = −1.40; *P*=0.14).

## 4. Discussion

The 2 traditionally mentioned together differential diagnoses with restrictive cardiac hemodynamic manifestations are CA and CP. Several clinical and imaging criteria have been described for the precise diagnosis and differentiation of these conditions [7, 8]. In the present investigation, we compared CMR features between 3 groups: CA, CP, and healthy controls. The main findings of our study were as follows:

1. All biventricular strain values were severely impaired in the CA and CP groups compared with the healthy controls.
2. The incidence of LVGLS ≤10% in the CA group was significantly higher than that of the CP group.
3. Compared with the patients with CP, LV strains were significantly reduced in the CA group. Among the RV strains, only RVGLS was meaningfully different between the 2 categories of patients.
4. In the CA group, LVGLS and GCS were significantly decreased at the basal level by comparison with the mid and apical parts. Moreover, LVGRS was diminished at both basal and mid-ventricular levels compared with the apex.
5. In our research, with a mean follow-up of 15.5 months, the mortality rate was 84.6% in the subjects with CA and 5.3% in those with CP.

Distinguishing between CA and CP is a challenging but vital process that affects patient survival [8]. The therapeutic options are revolutionized for CA, especially if diagnosed early. Moreover, CP is a curable disease by pericardiectomy, and the sooner the diagnosis of CA and CP is established, the better the outcome is [7, 22–24].

Detecting and classifying myocardial dysfunction in patients with CA before obvious clinical symptoms of heart failure are needed to find CA patients who benefit from autologous stem cell transplantation and high-dose chemotherapy to improve prognosis [7, 8, 22, 25, 26]. Strain analysis can successfully detect subtle functional impairments associated with several diseases such as CA and CP. In addition to conventional diagnostic methods, the ventricular strain measurement may be a beneficial diagnostic aid in differentiating between these 2 conditions (Figures 2(e) and 2(f)).

We analyzed myocardial FT parameters in 3 groups of patients: CA, CP, and healthy controls. Compared with the healthy control group, biventricular strain values, comprising GLS, GCS, and GRS, were severely impaired in both CA and CP groups.

Bhatti et al. showed that LVGLS was affected earlier than radial and circumferential strain in 46 patients with multiple myeloma with suspected CA [27]. They included a subgroup of patients with multiple myeloma who still had normal LV wall thickness with biopsy-proven CA, indicating early stages of the disease, while we examined patients with relatively advanced CA and apparent cardiac involvement in our CMR study. It seems that in the advanced stages of CA, all ventricular strains are severely reduced. We suppose that further studies on groups of amyloidosis patients concerning cardiac involvement severity may be of great interest.

The main finding of our study was that all global LV strain values demonstrated a meaningful decline in the CA group by in comparison with the CP group. Previous studies have revealed abnormal circumferential deformation, torsion, and untwisting velocity in CP accompanied by preserved GLS [28, 29]. We found that 68% (13/19) of the subjects with CA had LVGLS ≤10%, while only 5% (1/19) of the cases with CP showed the stated finding (*P* < 0.001). Therefore, it is logical to consider LVGLS as a powerful marker for differentiating between these disease classes. A previous study demonstrated that abnormal LV deformation was related to coronary microvascular dysfunction, which has a more prominent impact on the longitudinal function of the heart. Additionally, there is a link between impaired GLS and worse outcomes in patients with amyloid light chain and thus may help to predict prognosis in CA patients [22].

In our investigation, RVGCS and RVGRS impairment was similar between the CA and CP groups, while RVGLS was significantly lower in the CA patients compared with the CP subjects. In other words, only RVGLS had a significant difference in the CA group by comparison with the CP group. In CA patients, RV involvement happens in more advanced stages of the disease. Similar to our results, in another study, impaired RV-free wall longitudinal strain in patients with CA was reported [30]. We postulated that CP patients might have preserved RVGLS compared with CA cases and that it could be utilized as a CMR marker. Nonetheless, more research is needed in this regard.

We also demonstrated LV apical sparing in strain values in the subgroup of patients with CA. In previous investigations, similar to our current work, the pattern of LV apical sparing in strain values was also observed in cases with CA. The preservation of LV apical longitudinal strain in CA may be related to low amyloid deposition in this region compared with the base, and it is a sensitive and specific marker in CA patients [29]. Interestingly, Moñivas Palomero et al. described an apical sparing pattern in the RV as previously described in the LV [30]. Singh et al. mentioned that the accuracy of apical sparing in strain values for the diagnosis of amyloid light chain was reduced in patients with chronic kidney disease [31].

In our investigation, as in previous works, the prognosis of patients with CA was much worse than that of patients with CP. Approximately, 85% of our cases with CA died within an average of 15.5 months, indicating the importance of the early diagnosis of these patients. On the other hand, the early diagnosis of patients with CP is of utmost importance because, with timely surgical intervention, most patients survive and return to routine daily life.

### 4.1. Limitations

The major limitation of our study was its relatively small sample size within each group of CP and CA, precluding the extraction of a cutoff for strain values to differentiate between these categories of disease. Novel parametric mapping was not included in our study, and planning research with the inclusion of mapping techniques is helpful. Designing studies in the early stages of the disease is essential. Moreover, evaluation of the inter and intraobserver variability was not part of our study.

## 5. Conclusion

FT-CMR is a novel noninvasive method to detect insidious myocardial disorders. In the present study, cardiac deformation parameters were declined significantly in both groups of CA and CP compared with healthy controls. Biventricular GLS was meaningfully reduced in CA subjects. Strain analysis, especially in the longitudinal direction, is an auxiliary finding to differentiate CA from CP subjects.

## Figures and Tables

**Table 1:** Demographic and CMR parameters of the study population.

Variable	Age (mean ± SD)	Gender (male) %	
CA	57 ± 10	68.4%	
CP	51 ± 17	73.7%	
Control	32 ± 4	50%	

Study group	CA	CP	Control

Frequency (*n*)	19	19	28

CMR parameter	Mean ± SD	Mean ± SD	Mean ± SD

LVGLS%	8 ± 3	13 ± 2.7	18 ± 1.4
LVGCS%	10.5 ± 3.79	15 ± 3.2	18.7 ± 2.18
LVGRS%	17.5 ± 8.89	31 ± 12.3	40 ± 7.8
RVGLS%	14.6 ± 5.70	19.4 ± 5.90	23.6 ± 4.48
RVGCS%	9.9 ± 2.92	9.2 ± 4.00	17.7 ± 1.79
RVGRS%	16 ± 5.3	14.2 ± 5.96	31.1 ± 7.75
LVEF%	38 ± 11.9	52.5 ± 6.63	58 ± 2.7
LVEDVI (ml)	82.5 ± 33.99	64.3 ± 17.76	79.9 ± 2.94
LVESVI (ml)	52.6 ± 32.94	29.9 ± 6.64	34.5 ± 2.82
RVEF%	39 ± 12.0	47 ± 7.8	56 ± 2.2
RVEDVI (ml)	70.9 ± 25.40	71.2 ± 20.08	63.6 ± 3.00
RVESVI (ml)	43.3 ± 18.25	36.8 ± 12.27	28.8 ± 2.29
IAS thickness	5.8 ± 1.53	1.4 ± 0.2	1.4 ± 0.19
LV mass index	92.8 ± 25.81	57 ± 10.2	54.9 ± 8.57
LA area (cm^2^)	27.4 ± 7.24	24 ± 3.4	18 ± 1.7
RA area (cm^2^)	25.3 ± 4.10	18 ± 2.3	16.8 ± 1.60

CA: cardiac amyloidosis, CP: constrictive pericarditis, LV: left ventricle, RV: right ventricle, EF: ejection fraction, GLS: global longitudinal strain, GCS: global circumferential strain, GRS: global radial strain, CI: confidence interval, EDVI: end-diastolic volume index, ESVI: end-systolic volume index, IAS: interatrial septum, LA: left atrium, and RA: right atrium.

**Table 2:** The results of the post hoc Bonferroni test.

Dependent variables	Group 1	Group 2	Mean difference (95% CI)	*P* value
LVEF	Normal	CA	20.01 (14.64–25.38)	<0.001
	Normal	CP	5.70 (0.34–11.07)	0.03
	CP	CA	14.30 (20.16–8.45)	<0.001

RVEF	Normal	CA	16.46 (10.84–22.08)	<0.001
	Normal	CP	8.20 (2.58–13.82)	0.002
	CP	CA	8.26 (2.13–14.39)	0.005

LVGLS	Normal	CA	10.00 (8.30–11.71)	<0.001
	Normal	CP	5.05 (3.35–6.76)	<0.001
	CP	CA	4.95 (3.09–6.81)	<0.001

LVGCS	Normal	CA	8.19 (6.00–10.39)	<0.001
	Normal	CP	3.44 (1.25–5.64)	0.001
	CP	CA	4.75 (2.35–7.15)	<0.001

LVGRS	Normal	CA	22.28 (15.30–29.28)	<0.001
	Normal	CP	9.24 (2.26–16.25)	0.005
	CP	CA	13.04 (5.41–20.67)	<0.001

RVGLS	Normal	CA	8.98 (5.13–12.84)	<0.001
	Normal	CP	4.22 (0.37–8.08)	0.02
	CP	CA	4.76 (0.55–8.97)	0.02

RVGCS	Normal	CA	7.80 (5.69–9.92)	<0.001
	Normal	CP	8.46 (6.35–10.58)	<0.001
	CP	CA	−0.65 (−2.97–1.65)	1.00

RVGRS	Normal	CA	15.09 (10.25–19.95)	<0.001
	Normal	CP	16.83 (11.98–21.69)	<0.001
	CP	CA	−1.73 (−7.03–3.56)	1.00

CA: cardiac amyloidosis, CP: constrictive pericarditis, LV: left ventricle, RV: right ventricle, EF: ejection fraction, GLS: global longitudinal strain, GCS: global circumferential strain, GRS: global radial strain, and CI: confidence interval.

## Data Availability

The datasets generated during the current research are available from the corresponding author on reasonable request.
